# Preclinical Determinants of Drug Choice under Concurrent Schedules of Drug Self-Administration

**DOI:** 10.1155/2012/281768

**Published:** 2012-11-28

**Authors:** Matthew L. Banks, S. Stevens Negus

**Affiliations:** Department of Pharmacology and Toxicology, Virginia Commonwealth University, P.O. Box 980613, Richmond, VA 23298, USA

## Abstract

Drug self-administration procedures have played a critical role in the experimental analysis of psychoactive compounds, such as cocaine, for over 50 years. While there are numerous permutations of this procedure, this paper will specifically focus on choice procedures using concurrent schedules of intravenous drug self-administration. The aims of this paper are to first highlight the evolution of drug choice procedures and then review the subsequent preclinical body of literature utilizing these choice procedures to understand the environmental, pharmacological, and biological determinants of the reinforcing stimulus effects of drugs. A main rationale for this paper is our proposition that choice schedules are underutilized in investigating the reinforcing effects of drugs in assays of drug self-administration. Moreover, we will conclude with potential future directions and unexplored scientific space for the use of drug choice procedures.

## 1. The Evolution of Drug Choice Procedures

Drug self-administration procedures have played a critical role in the experimental analysis of psychoactive compounds, such as cocaine, for more than 50 years. In general, preclinical drug self-administration procedures are utilized for two main scientific purposes. One purpose is in abuse liability testing of psychoactive compounds for potential scheduling as controlled substances by the Drug Enforcement Agency, and there are already excellent reviews on the use of drug self-administration procedures for this purpose, see [1, 2]. The other main purpose of drug self-administration procedures is in understanding the pharmacological, environmental and biological determinants of drug-taking behavior as a model of drug addiction. This paper will focus on the use of concurrent-choice schedules of drug self-administration to address this latter purpose.

Although there are numerous permutations of drug self-administration procedures, all use the classic 3-term contingency of operant conditioning to investigate the stimulus properties of drugs [3]. This 3-term contingency can be diagrammed as follows:

(1) $$\begin{array}{c} S^{D}\to R\to S^{C}, \end{array}$$

where *S* ^*D*^ designates a *discriminative stimulus*, *R* designates a *response* on the part of the organism, and *S* ^*C*^ designates a *consequent stimulus*. The arrows specify the contingency that, in the presence of the discriminative stimulus *S* ^*D*^, performance of the response *R* will result in delivery of the consequent stimulus *S* ^*C*^. As a simple and common example from a preclinical laboratory, a rat implanted with a chronic indwelling catheter might be connected to an infusion pump containing a dose of a psychoactive drug and placed into an experimental chamber that contains a stimulus light and a response lever. Contingencies can be programmed such that, if the stimulus light is illuminated (the discriminative stimulus), then depression of the response lever (the response) will result in delivery of a drug injection (the consequent stimulus). Conversely, if the stimulus light is not illuminated, then responding does not result in the delivery of the drug injection. Under these conditions, subjects typically learn to respond when the discriminative stimulus is present. Consequent stimuli that increase responding leading to their delivery are operationally defined as *reinforcers*, whereas stimuli that decrease responding leading to their delivery are defined as *punishers*. The contingencies that relate discriminative stimuli, responses, and consequent stimuli are defined by the schedule of reinforcement [4]. 

 The first published reports of intravenous drug self-administration used exactly this type of single-response procedure described above to examine morphine self-administration in morphine-dependent rats [5, 6]. Furthermore, these seminal studies demonstrated three principles that have been commonly observed in single-response drug self-administration procedures ever since. First, these studies demonstrated that intravenous morphine could maintain schedule-appropriate rates and patterns of responding leading to its delivery, indicating that morphine functioned as a reinforcer. Second, this single-response procedure produced a bitonic, “inverted U shaped” dose-effect function relating the unit dose of morphine in each injection to measures of rate (either rates of responding or injection delivery). Thus, maximal rates of self-administration were maintained by intermediate morphine doses, and lower rates were maintained not only by lower morphine doses, but also by higher doses. Importantly, this pattern of responding indicates a dissociation between rates of self-administration maintained by a given consequent stimulus and the reinforcing efficacy of that stimulus [7, 8], because if the research subject is given a choice between a lower and higher dose of the same drug, the subject will almost always choose the higher drug dose indicating that the higher drug dose is the preferred or more efficacious reinforcer [9, 10]. Why would rates of drug self-administration decrease as dose increases above some apparent optimal level? As is often the case in other pharmacology domains, the presence of a bitonic dose-effect function indicates that multiple and/or opposing drug effects are being integrated into a common dependent variable. For example, measures of self-administration rate can be influenced not only by the reinforcing effects of a drug (which would have the effect of increasing rates), but also by other effects of the self-administered drug that can either increase or decrease rates (e.g., effects that improve or impair motor competence or information processing). These other drug effects will be collectively referred to as “reinforcement-independent rate-altering effects” in this paper to distinguish them from reinforcing effects, and one goal of more recently developed procedures is to dissociate reinforcing drug effects from reinforcement-independent rate-altering effects. 

The third and final principle revealed by these early studies was that rates of morphine self-administration could be altered by treatment with other drugs. These effects were interpreted to suggest treatment effects on drug reinforcement (and by extension, to provide evidence regarding mechanisms of drug reinforcement). However, just as self-administration rates can be influenced by multiple effects of the self-administered drug, so these rates can also be influenced by multiple effects of a treatment drug (or of any other experimental manipulation, such as a lesion or genetic modification) [11, 12]. More specifically, these experimental manipulations can alter rates of self-administration not only by changing the reinforcing effects of the self-administered drug, but also by changing the reinforcer-independent rate-altering effects of the self-administered drug, or by producing its own reinforcement-independent rate-altering effects. Overall, these early studies illustrated the promise of drug self-administration as a model of drug addiction, but they also provided a glimpse of the challenges to interpretation of rate-based measures generated by single-response procedures.

Since the early 1960s, preclinical drug self-administration research has flourished, and techniques for intravenous drug self-administration were rapidly extended to studies with other drug classes and in other species of experimental subjects [13, 14]. However, over the decades, drug reinforcement research has evolved along three divergent paths. One path of self-administration research has retained the use of single-response procedures initially used by Weeks and colleagues [5, 6] and utilized more demanding and complex schedules of reinforcement, such as progressive-ratio and second-order schedules, than the simple fixed-ratio schedules used by Weeks and colleagues. In general, studies using these approaches have demonstrated that numerous drug classes can maintain rates and patterns of responding consistent with the hypothesis that drugs can function as reinforcers [15]. However, these approaches have been less successful in generating dependent measures that clearly dissociate reinforcing drug effects from reinforcement-independent rate-altering drug effects. To highlight the prevalence of single-response procedures in the current drug self-administration literature, we used the keyword “self-administration” in PubMed on April 11, 2012, to retrieve the 50 most recent preclinical studies using intravenous drug self-administration procedures as the primary independent variable. This “snapshot” of the literature revealed that 15 of the 50 most recent studies used a single-response drug self-administration procedure. 

 A second path of self-administration research has retained the simple fixed-ratio schedules utilized by Weeks and colleagues on one response lever, but incorporated rudimentary aspects of choice by introducing an “inactive” response option in addition to the “active” drug option. For example, an early study by Pickens and Thompson [16] used a two-lever self-administration procedure in which responding on one “active” lever produced intravenous cocaine delivery under an FR1 schedule of reinforcement, whereas responding on a second “inactive” lever had no scheduled consequences. Schedule-appropriate responding was maintained exclusively on the “active” lever, and when the contingencies on the two levers were reversed, rats rapidly reallocated their responding to the newly “active” lever. In our snapshot analysis of the current self-administration literature, the majority of drug self-administration studies (32 out of 50) used an “inactive” manipulandum. Differential rates of responding on “active” and “inactive” manipulanda are useful for investigating the reinforcing effects of consequent stimuli associated with the “active” manipulandum; however, the utility of this simple type of choice procedure is limited for at least two main reasons. First, although “active/inactive-response” procedures technically employ a concurrent schedule capable of generating measures of response allocation and choice, such measures are rarely computed or reported. Rather, investigators more commonly report measures of response or reinforcement rate on the “active” manipulandum as if it were the only response option available, and such rate-based measures of drug reinforcement are vulnerable to all the reinforcement-independent rate-altering drug effects described above. Second, baseline rates of behavior on the “active” and “inactive” manipulanda are normally vastly different, with rates on the “inactive” manipulandum being very low. As a result, data on “inactive” responding are primarily useful for only detecting reinforcement-independent rate-*increasing* effects. However, because “inactive” rates are already low, they are insensitive to reinforcer-independent rate-*decreasing* effects of experimental manipulations. This is a critical issue, because most drug self-administration studies are designed to evaluate the ability of experimental manipulations, such as pharmacological, environmental, or genetic variables, to decrease drug reinforcement as indicated by decreases in drug self-administration rates. Thus, procedures that use “active” and “inactive” manipulanda are not much different than the single-response self-administration procedures described above. 

**Figure 1:**
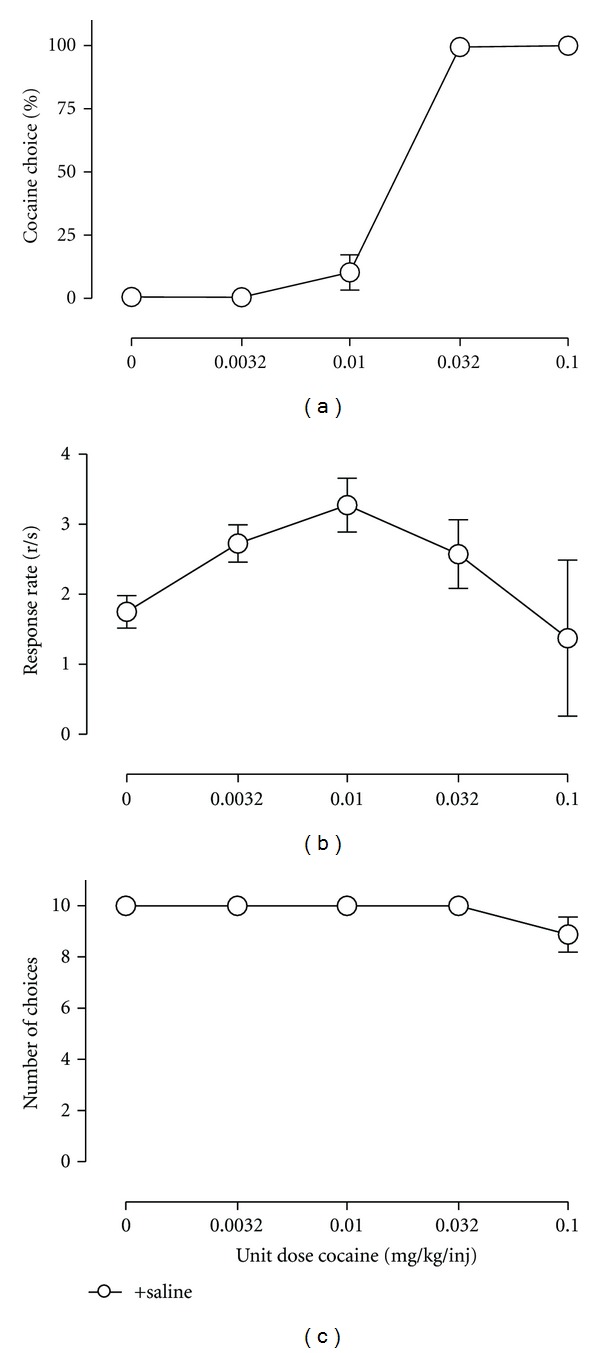
Baseline choice between different doses of cocaine (0–0.1 mg/kg/injection) and food pellets in rhesus monkeys (*n* = 4) under a concurrent FR10 : FR100 schedule of cocaine injections and food availability. Abscissae: unit dose of cocaine in milligrams per kilogram per injection. Top ordinate: percent cocaine choice. Middle ordinate: rates of responding in responses per second. Bottom ordinate: number of choices completed. All points represent mean data SEM obtained during the last 3 days of saline treatment. These unpublished data demonstrate two key observations from choice procedures. First, cocaine choice increases in a monotonic function as the unit cocaine dose increases. Second, while rates of responding display the prototypic, inverted-U-shaped dose-effect function, rates of responding are not predictive of cocaine choice, nor are rates of responding predictive of the number of choices completed per component.

**Figure 2:**
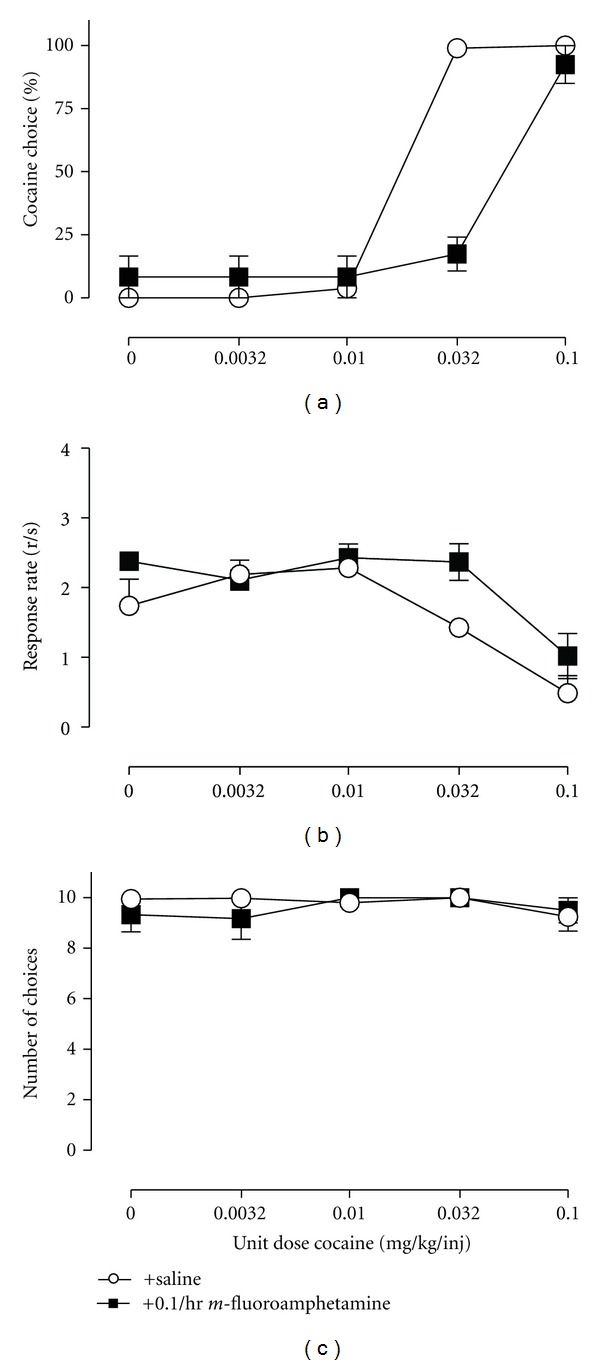
Effects of chronic intravenous *m*-fluoroamphetamine (0.1 mg/kg/hr) administration on choice between cocaine and food in rhesus monkeys (*n* = 4). Abscissae: unit dose of cocaine in milligrams per kilogram per injection. Top ordinate: percent cocaine choice. Middle ordinate: rates of responding in responses per second. Bottom ordinate: number of choices completed. All points represent mean data SEM obtained during the last 3 days of *m*-fluoroamphetamine treatment. These published data [17] demonstrate that experimental manipulations can selectively decrease cocaine choice without also decreasing rates of responding and the number of choices completed. This profile would be considered ideal for a candidate medication to treat cocaine dependence.

**Figure 3:**
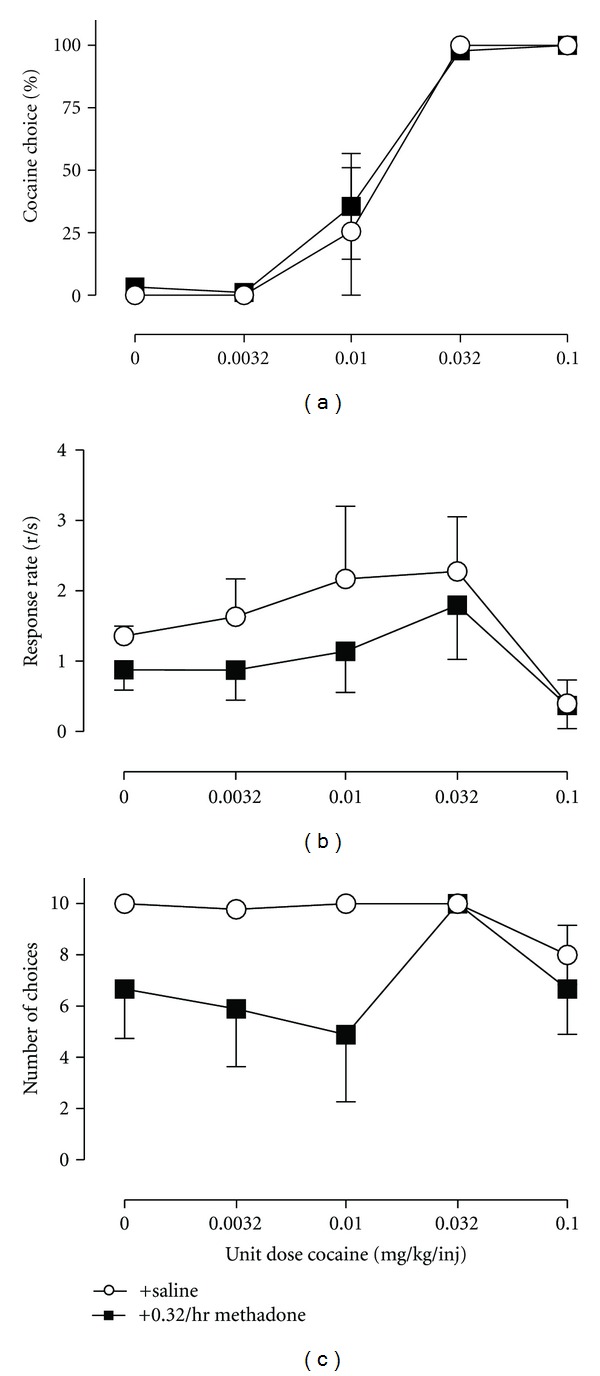
Effects of chronic intravenous methadone (0.32 mg/kg/hr) administration on choice between cocaine and food in rhesus monkeys (*n* = 3). Abscissae: unit dose of cocaine in milligrams per kilogram per injection. Top ordinate: percent cocaine choice. Middle ordinate: rates of responding in responses per second. Bottom ordinate: number of choices completed. All points represent mean data SEM obtained during the last 3 days of methadone treatment. These published data [18] demonstrate that experimental manipulations can selectively decrease rates of responding and the number of choices completed without decreasing cocaine choice.

The third and least common path of drug self-administration research has used concurrent schedules in which responding is maintained on two or more manipulanda by two or more motivationally relevant consequent stimuli. For example, responding on one manipulandum might result in delivery of a particular drug dose, and responding on a different, concurrently available manipulandum might result in delivery of a different dose of the same drug, a different drug, or a qualitatively different consequent stimulus such as food (Figure 1). These procedures are often referred to as “choice” procedures, because subjects allocate their behavior, or “choose,” between the available consequent stimuli, and the relative reinforcing effects of drug in comparison to an alternative are derived from measures of behavioral allocation (or “drug choice”) rather than behavioral rate. As with any other type of self-administration procedure, the self-administered drug or other experimental manipulations might also influence overall self-administration rate by producing reinforcement-independent rate-altering effects; however, the impact of these other effects on choice measures of drug reinforcement can be minimized by appropriate use of manipulanda, discriminative, and alternative reinforcing stimuli, and schedules of reinforcement. A specific example of this dissociation is shown in Figure 1. Cocaine versus food choice increases as the unit cocaine dose increases; however, rates of responding display the prototypic inverted-U shaped dose-effect function. Moreover, choice procedures generate distinct measures of behavioral allocation and behavioral rate that permit dissociation of reinforcing effects from reinforcement-independent rate-altering effects. For example, an experimental manipulation that decreases reinforcing efficacy of a drug might be expected to reduce drug choice but increase choice of the alternative and produce no net change in overall reinforcement rates. A specific example of this selective effect from the literature is shown in Figure 2 examining cocaine versus food choice during chronic treatment with the dopamine (DA)-selective releaser *m*-fluoroamphetamine [17]. Conversely, a manipulation that produces reinforcement-independent rate-altering effects (e.g., motor impairment) might be expected to reduce overall reinforcement rates without altering drug choice. An example of this specific effect from the literature is shown in Figure 3 examining cocaine versus food choice during chronic treatment with the mu-opioid agonist methadone [18]. These distinct dependent measures of reinforcing effects (represented in measures of drug choice) and reinforcement-independent rate-altering effects (represented in measures of overall self-administration rates) are analogous to the use of concurrent schedules in the closely related field of drug discrimination research to generate dependent measures that permit dissociation of discriminative stimulus effects (represented in measures of drug-appropriate responding) from discrimination-independent rate-altering effects (represented in measures of overall rates of responding or reinforcement). Despite this apparent advantage, our PubMed search indicated that only 3 of the 50 most recent IV drug self-administration studies used a concurrent schedule of reinforcement. This paucity of research with concurrent schedules in research on the reinforcing stimulus effects of drugs stands in striking contrast to the almost exclusive use of concurrent schedules in drug discrimination research and suggests that concurrent schedules are underutilized in studies of drug self-administration [19].

Although choice procedures have been underutilized, their value has long been appreciated. As noted above, the earliest studies of *intravenous* drug self-administration used single-response procedures, but these studies were predated by choice studies in which drug was delivered by other routes of administration. For example, more than two decades before the studies by Weeks and colleagues, Spragg evaluated choice between intramuscular morphine and fruit in morphine-dependent chimpanzees and demonstrated that choice was largely influenced by the state of morphine withdrawal (such that morphine withdrawal was associated with increased probability of morphine choice) [20]. Similarly, Nichols and colleagues established responding for oral morphine in rats and found that morphine withdrawal increased choice of morphine over water [21]. Intravenous drug delivery subsequently gained prominence in drug self-administration research because it promotes a rapid onset of drug action that facilitates learned contingencies between responding and drug delivery. However, the rise of intravenous drug self-administration was also accompanied by a growing reliance on single-response and “active/inactive”-response procedures, perhaps because the limited lifespan of intravenous catheters selected for procedures that require the least initial training. Nonetheless, the use of choice procedures persisted, especially in studies of oral drug self-administration [13, 22] and in a small but steady series of intravenous drug self-administration studies. The goal here is to review the history and major findings of research on intravenous drug choice.

Before proceeding, two other points are worthy of mention. First, although choice procedures are sparingly used in *preclinical* studies of drug reinforcement, they have emerged as the standard approach in *clinical* studies of drug reinforcement [23, 24]. Consequently, increased preclinical use of choice procedures might facilitate translational research on drug reinforcement. Second, scientific interest in drug reinforcement derives in large part from its presumed role in drug addiction, and drug addiction can be defined as a disorder of choice and behavioral allocation [25, 26]. Moreover, 5 of the 7 diagnostic criteria for substance dependence in the revised fourth edition of the Diagnostic and Statistical Manual (DSM) of Mental Disorders are defined by allocation of behavior towards procurement and use of the substance compared to other behavior maintained by nondrug and presumably more adaptive alternative reinforcers [27]. In the fifth edition of the DSM that is still under development, 6 of the 11 diagnostic criteria are defined by behavioral allocations toward the procurement and use of the substance [28]. Thus, addiction implies excessive drug choice at the expense of more adaptive behaviors. The pharmacological, environmental, and genetic determinants that influence drug choice and contribute to drug addiction can be directly studied using choice procedures.

## 2. Determinants of Drug Choice

### 2.1. Overview

While the first drug choice procedure was published in 1940 [20] by Spragg it was not until 1972 that the first intravenous drug choice procedure was published, approximately 10 years after intravenous drug self-administration procedures were introduced [5]. In their seminal study, Findley et al. [29] examined the effects of dependence and withdrawal on secobarbital and chlordiazepoxide preference. Since 1972, there have been 66 publications examining the determinants of drug choice, and these publications are summarized in Table 1. The predominant drugs examined have been cocaine (80%) and heroin (15%). Furthermore, nonhuman primates have been the predominant research subjects (81%) utilized in these studies, with rhesus monkeys (70%) being the most commonly used species. The results and implications of this literature will be reviewed in more detail below. 

### 2.2. Choice between Drug and Itself

#### 2.2.1. Effect of Dose

One of the first fundamental research questions to be answered was whether drug choice was dose-dependent. This question was important in determining whether drug choice varied independently of rates of responding as drug dose increased, such that, choice would increase and response rates decrease as a function of increasing drug doses. Although this specific experimental question has only been explicitly examined using intravenous cocaine as the reinforcer and rhesus monkeys as the research subjects, animals almost always choose the larger drug dose [9, 30–33]. Furthermore, in the manuscripts that did report rates of responding, there was no systematic relationship between cocaine choice and rates of cocaine-maintained responding [30, 34]. Overall, this body of literature supports the conclusion that drug choice is dose-dependent and that drug choice may be less sensitive than single-response procedures to reinforcer-independent rate-altering drug effects.

#### 2.2.2. Effect of Temporal Parameters of Reinforcer Delivery

Another fundamental question to be answered was whether drug choice was sensitive to manipulations in the delivery of the drug. In general, when equal drug doses are available as the consequence for two response options, research subjects will allocate their behavior equally between the two response options. However, if the infusion rate was to be varied between the two response options, subjects will almost exclusively choose the dose associated with the shorter (faster) infusion rate [32, 35, 36]. The delay between response and drug delivery is a related variable that has been manipulated in choice studies [31, 37]. For example, Woolverton and Anderson [37] systematically varied the delay between completing the response requirement and delivery of the intravenous drug injection. When the delay was 0 sec and the choice was between a low (0.025 mg/kg/injection) and high (0.05 mg/kg/injection) unit cocaine dose, the subjects almost exclusively chose the high cocaine dose. However, increasing delays in the delivery of the high cocaine dose produced a monotonic decrease in high cocaine dose choice and a reciprocal increase in choice of the alternative low cocaine dose. Overall, these data support the notion that drug choice is highly sensitive to manipulations that affect the timing or probability of reinforcer delivery and that subjects prefer reinforcers to be delivered quickly and with no delay. 

#### 2.2.3. Effect of Schedule of Reinforcement

Finally, a third fundamental question to be addressed was whether the programmed schedules of reinforcement influenced drug choice. For example, when rhesus monkeys were given a choice between identical cocaine doses, but the probability of reinforcement was decreased such that every other FR5 (probability 50%) or every fourth FR5 (probability 25%) completion resulted in delivery of the cocaine injection on one of the response options, monkeys consistently chose the response option associated with the higher probability of reinforcement [38]. Several laboratories have examined the effects of schedule manipulations under concurrent variable-interval (VI): VI schedules. Under a VI schedule of reinforcement, the first response after a variable amount of time has passed results in presentation of the reinforcer. The variability in time is anchored at some programmed time interval by the investigator such that, on average, the interval of reinforcement is the anchored time, for example 600 sec. Most of the studies have examined cocaine versus cocaine choice [30, 33, 39, 40], but a few have also examined other drugs such as the mu-opioid agonists alfentanil [41] or remifentanil [42] and the barbiturate methohexital [41, 42]. In general, these studies have been used to demonstrate that drug self-administration procedures adhere well to the predictions of the matching law, which posits that the allocation of behavior between two response options will match the frequency of reinforcement associated with those options [30, 33, 39–41]. Moreover, as predicted, subjects will chose reinforcers delivered under shorter versus longer VI schedules. In contrast to concurrent VI: VI schedules, only one published study has examined the effects of manipulating the response requirement under a concurrent fixed-ratio (FR): FR schedule of choice between a drug and itself [31]. In this study, only one of the three monkeys was sensitive to FR manipulations such that increases in the FR requirement decreased choice of the higher unit cocaine dose and produced a reciprocal increase in choice of the lower unit cocaine dose. Thus, choice behavior maintained under concurrent FR: FR schedules appears to be more quantal than choice behavior maintained under current VI: VI schedules. Overall, this body of the literature suggests that subjects prefer schedules of reinforcement that produce the higher probability of reinforcement. 

### 2.3. Choice between Drug and Alternatives

#### 2.3.1. Behavioral Economic Considerations

 One method to understand how concurrently available reinforcers interact is to apply conceptual frameworks employed by behavioral economics [43, 44]. Based on economic theories, concurrently available reinforcers can interact in one of three ways [44, 45]. First, concurrently available reinforcers can function as *substitutes*; such that as the price of one reinforcer increases, choice of that reinforcer decreases and is replaced by choice of the substitute. The perfect substitute for a commodity is itself, so that studies summarized above that considered choice between a drug and itself could be conceptualized as choice between substitutes. However, different commodities can also function as economic substitutes for each other. For example, potato chips and pretzels can function as substitutes, such that as the price of potato chips increases, consumption of potato chips decreases and consumption of pretzels increases. Secondly, concurrently available reinforcers can function as *complements*; under this condition, as price of one reinforcer increases, choice of that reinforcer, and choice of a complement also decreases. For example, peanut butter and jelly can function as complements, and as the price of peanut butter increases, choice of both peanut butter and jelly decreases. Finally, concurrently available reinforcers can function as *independents*, such that changes in the price and consumption of one reinforcer would have no effect on choice of an independent reinforcer. For example, peanut butter and shoes typically function as independents, such that as the price of peanut butter increases and choice of peanut butter decreases, and consumption of shoes is unlikely to change. The interaction between two concurrently available reinforcers will be an important consideration in the following sections. In general, alternative reinforcers used in studies of drug choice are expected to function as substitutes, but this is an empirical question. 

#### 2.3.2. Drug versus Other Drugs

To ascertain the relative reinforcing efficacy of two different drugs in maintaining behavior, a choice procedure could be programmed. As stated earlier, a significant advantage of choice procedures is that the primary dependent measure (behavioral allocation or choice) is less confounded by reinforcement-independent rate-altering drug effects. Critical factors to consider when assessing the relative reinforcing efficacy between two different drugs are dose, pharmacokinetics, and pharmacodynamics. The entire literature on choice between two drugs has employed cocaine versus “drug X” choice procedures, with “X” being the dopamine (DA) uptake inhibitor methylphenidate [10, 31], the sodium channel blocker procaine [46], the monoamine releaser cathinone [47], DA agonists [48], nicotine [49], the DA uptake inhibitor 2*β*-propanoyl-3*β*-(4tolyl)-tropane (PTT) [50], or the mu-opioid agonists remifentanil [51] and heroin [52]. In general, these studies have reported that as dose of the alternative drug reinforcer increased, cocaine choice decreased. Such results are consistent with the conclusion that the alternative drug functioned as a substitute for cocaine. However, there were two notable exceptions. When choice was between cocaine versus procaine [46] or cocaine versus nicotine [49], cocaine choice predominated despite increasing doses of procaine or nicotine or decreasing doses of cocaine. Moreover, only one study has explicitly examined whether two drugs function as substitutes, complements, or independents. In monkeys choosing between cocaine and the mu-opioid agonist remifentanil, remifentanil was found to function as a behavioral economic substitute for cocaine [51]. Overall, drug versus drug choice procedures can be useful for assessing the relative reinforcing efficacy of two different compounds and this procedure may hold utility in abuse liability testing. 

#### 2.3.3. Drug versus Nondrug Reinforcers

Analysis of the choice studies in Table 1 revealed that 61% (41/67) used a nondrug alternative reinforcer with 93% (38/41) of these studies using food and 7% (3/41) using saccharin or sucrose as the alternative. In the first drug versus nondrug choice procedure, rhesus monkeys were allowed to choose between cocaine injections and food in a closed economy, such that no other food source was available outside of the choice procedure [53]. Over the 8 experimental days, monkeys almost exclusively choose cocaine over food despite body weight decreases of 6 to 10% over the course of the 8 days. More recent studies have utilized other experimental designs to evaluate the effects of multiple cocaine doses versus food using a within-session choice procedure [17, 54–56]. For example, Figure 1 demonstrates that a complete cocaine versus food dose-effect function can be determined within a single daily experimental session. Cocaine choice increased in a monotonic function demonstrating that the relative reinforcing efficacy of cocaine versus food is dose-dependent. Furthermore, this monotonic increase in cocaine choice was in contrast to rates of responding, which displayed the prototypic inverted U-shaped dose-effect function. Thus, the example shown in Figure 1 clearly demonstrates the dissociation between using dependent measures of behavioral allocation (choice) and behavioral rate discussed above as a main rationale for the use of drug choice procedures. 

As was evident in drug versus drug choice procedures described above, the magnitude of the alternative nondrug reinforcer, programmed schedule consequences, and reinforcement delay were also important independent variables that could impact drug choice [37, 55, 57–60]. For example, increasing the magnitude of the alternative food reinforcer was shown to decrease cocaine choice [55, 57]. In another example, heroin versus food choice was decreased by increasing the intertrial interval [60]. These results suggest that under economic conditions where access to reinforcers was restricted, baboons choose food over a low dose of heroin. Moreover, if the reinforcing value of food was decreased by providing supplemental access to food before the cocaine versus food choice procedure, cocaine choice increased [55, 59]. 

While most of the drug versus nondrug alternative choice procedures discussed so far used nonhuman primates as research subjects, there is a small, but growing body of literature of drug versus food choice procedures in rodents. For example, a recent study by Thomsen et al. [61] established a within-session cocaine dose-effect versus ensure choice procedure similar to the within-session cocaine dose-effect versus food choice procedure shown in Figure 1. Other rodent studies have demonstrated that introduction of an alternative nondrug reinforcer such as a glucose/saccharin solution [62] or food [63, 64] will attenuate cocaine or methamphetamine choice. In contrast to these other rodent studies, rats choosing between cocaine injections and 0.2% saccharin never chose cocaine over the saccharin solution [65]. This later result suggests that 0.2% saccharin is a very strong and highly preferred reinforcer in rats. Overall, this body of literature demonstrates that nondrug reinforcers can decrease drug choice, but that reinforcer selection, reinforcer magnitude, delay of reinforcement, and reinforcer access conditions are all key independent variables to be considered in drug versus nondrug choice procedures.

#### 2.3.4. Drug versus Compound Consequent Stimuli

In general, two categories of studies have investigated drug choice in the context of another compound consequent stimulus. One category has involved assessment of choice involving drug plus another putative reinforcer (e.g., drug + drug or drug + food). For example, when contingencies were programmed such that one response option produced a high heroin dose and a second response option produced a lower heroin dose delivered in combination with food, monkeys reallocated their behavior away from the high dose heroin towards lower heroin doses plus food [66]. Other studies have examined choice between (a) food and either cocaine alone or cocaine + mu-opioid agonist, or (b) choice between food and either mu agonist alone or delta-opioid + mu agonist [67–70] and, in general, reported that the relative reinforcing efficacies of these drug mixtures were additive. 

The other category has involved pairing one of the choices with a putative punisher, such as electric shock [71] or intravenous histamine [72, 73]. For example, Johanson [71] examined choice between cocaine alone versus cocaine + electric shock. In all of these studies, the punisher was effective in decreasing choice of the reinforce + punisher and increasing choice of the alternative reinforcer [71–73]. However, effects of punishment on drug choice could be mitigated by increasing the drug dose [71] associated with the punisher, decreasing the intensity of the punisher [72, 73], or increasing the delay between delivery of the reinforcer and delivery of the punisher [73]. Furthermore, pairing the punisher with the alternative reinforcer can also increase drug choice. For example, in a study of cocaine versus food choice, histamine injections paired with cocaine decreased cocaine choice, but if the histamine injections were paired with food, cocaine choice increased [72]. Moreover, these studies highlight the potential for cocaine use to be influenced by environmental contingencies that may govern choice of nondrug alternative reinforcers. Overall, these results highlight the utility of concurrent schedules of reinforcement to understand relatively sophisticated behaviors maintained by complex, compound consequent stimuli. Moreover, the use of choice procedures to understand abuse of multiple drugs, drug mixtures, and drug + other consequent stimuli is a scientific space that remains relatively unexplored. 

### 2.4. Other Factors Affecting Drug Choice

#### 2.4.1. Effect of Drug Dependence and Withdrawal

Most published studies examining effects of dependence and withdrawal on drug choice have focused on opioids [66, 74–78]. In contemporary experimental designs examining choice of mu agonists such heroin or the short-acting opioid remifentanil, the total amount of opioid available during daily choice sessions is sufficiently limited to prevent development of significant opioid dependence. Under these nondependent conditions, opioid choice can be effectively reduced by treatment with opioid antagonists like naloxone [76]. However, if opioid dependence is established by chronic noncontingent opioid treatment or by permitting supplemental daily access to contingent opioid self-administration, then drug effects on opioid choice change dramatically. So long as dependence is maintained by opioid agonist exposure, opioid choice during choice sessions is generally maintained (when the alternative is food, [76, 79]) or reduced in some subjects (when cocaine is the alternative, [78]). However, either spontaneous withdrawal or antagonist-precipitated withdrawal produce robust increases in opioid choice, and this withdrawal-associated increase in opioid choice can be blocked by opioid agonists such as methadone, which are effective maintenance medications for treatment of opioid addiction [75, 76, 78, 79]. Overall, then, opioid withdrawal in opioid-dependent subjects increases opioid choice, and opioid agonists that are effective maintenance medications for treatment of opioid addiction can block this withdrawal-associated increase in opioid choice.

 In contrast to the opioid dependence literature cited above, minimal research has been conducted examining the effects of dependence and withdrawal on drug choice maintained by other drug classes. Consistent with the opioid studies described above, Findley and colleagues [29] reported that withdrawal from the barbiturate secobarbital in secobarbital-dependent subjects increased choice of lower secobarbital doses versus food. However, similar results have not been demonstrated with cocaine. For example, Banks and Negus [80] evaluated cocaine versus food choice when subjects were exposed to and withdrawn from supplemental daily access to cocaine self-administration under conditions identical to those used to establish opioid dependence [76]. During supplemental cocaine access, daily cocaine intake increased more than 10-fold and was sufficient to disrupt performance during choice sessions; however, neither exposure to nor withdrawal from supplemental cocaine significantly altered cocaine versus food choice. The extent to which drug dependence and withdrawal alters drug choice of other drug classes, such as benzodiazepines, monoamine releasers, or N-methyl D-aspartate antagonists, remains to be elucidated. 

#### 2.4.2. Effect of Pharmacological Variables

 Another important body of literature has examined effects of pharmacological manipulations on drug choice, either to evaluate effects of candidate antiaddiction medications or to evaluate mechanisms of drug reinforcement. Effects of opioid agonists and antagonists on opioid choice in nondependent and opioid-dependent rhesus monkeys was described above, and additional studies have investigated potential mechanisms that may underlie withdrawal-associated increases in opioid choice. For example, opioid withdrawal functions as a stressor to activate endogenous release of the stress-related neurotransmitters dynorphin and corticotrophin releasing factor (CRF). This suggested that the hypothesis that either dynorphin acting at kappa-opioid receptors or CRF acting at CRF1 receptors might contribute to withdrawal-associated increases in opioid reinforcement; however, neither the kappa antagonist 5′-guanidinonaltrindole nor the CRF antagonist antalarmin was as effective as morphine in blocking withdrawal-associated increases in opioid choice [79]. 

A total of 10 studies have investigated pharmacological modulation of cocaine versus food choice. In studies examining candidate “agonist” medications for cocaine addiction, DA-selective monoamine releasers, such as d-amphetamine and phenmetrazine, significantly decreased cocaine choice, whereas mixed DA-serotonin (5HT) releasers or 5HT-selecitve releasers did not [17, 55, 81]. Importantly, these studies demonstrated a selective decrease in cocaine choice without also decreasing rates of behavior, and a representative example of this selective effect is shown in Figure 2 during treatment with the DA-selective releaser *m*-fluoroamphetamine. The atypical antipsychotic and DA D2 receptor partial agonist aripiprazole also decreased cocaine choice in rats after acute treatment, although this effect was not sustained during repeated treatment, and neither acute nor repeated aripiprazole altered cocaine choice in rhesus monkeys [61, 82]. Treatment with the benzodiazepine agonist diazepam also decreased cocaine choice in rats [83]. In contrast to these studies showing decreases in cocaine choice, cocaine choice was increased by treatment with a 5HT1A agonist, a kappa-opioid, agonist and high doses of dopamine receptor antagonists [55, 84–86]. Finally, other treatments that have failed to alter cocaine choice up to doses that suppress responding include methadone and lithium [18, 87]. Overall, this body of the literature suggests that drug choice is sensitive to both acute and chronic pharmacological manipulations, that pharmacological effects on drug choice can be dissociated from other drug effects, and that there is a clear need for more research in understanding the pharmacological mechanisms of drug choice.

One final point regarding the effects of pharmacological variables on drug choice is worth mentioning. An overarching rationale for preclinical studies investigating the pharmacological determinants of drug self-administration is the development of candidate medications to treat drug addiction. Moreover, a main goal of pharmacotherapy should be not only to *decrease *drug-taking behavior, but also to *reallocate* behavior to activities maintained by more adaptive reinforcers [88]. Thus, in preclinical studies that aspire to evaluate candidate medications for drug addiction, choice procedures can play a critical role in the preclinical drug development process to determine whether a given experimental manipulation produces this critical *reallocation* of behavior [19, 24]. Furthermore, human laboratory studies provide an additional critical step in drug development between animal studies and clinical trials, and human laboratory research to evaluate effects of candidate medications on drug self-administration relies exclusively on drug versus nondrug choice procedures [24]. Consequently, the use of choice procedures in preclinical studies may facilitate translation of results to choice procedures in human laboratory studies at this critical juncture in the drug development process, and existing data suggest excellent concordance between medication effects on drug choice in animals and humans [55, 85, 89–91]. 

#### 2.4.3. Effect of Other Environmental Variables

An emerging body of research has also addressed the degree to which non-pharmacological environmental variables might alter drug choice. In one example, monkeys were housed in a social context to establish a dominant-subordinate hierarchy to examine the effects of social rank on cocaine versus food choice [92]. One main rationale for this study was that the initial differences between dominant and subordinate monkeys in cocaine-maintained responding under a simple FR schedule disappeared over time as the cocaine self-administration history progressed. Under the concurrent FR: FR schedule of cocaine and food reinforcement, the differences between dominant and subordinate monkeys were recaptured. Furthermore, cocaine choice in socially housed monkeys was decreased by manipulations of environmental variables that were perceived to be enriching (increased cage size), whereas cocaine choice was decreased by environmental variables that were perceived to be stressful (exposure to rubber snake) [56]. Another study examined the effects of ambient temperature on choice between 3,4-methylenedioxymethamphetamine (MDMA) and food in rhesus monkeys [93]. Compared to room temperature, cool ambient temperatures decreased MDMA choice and warm ambient temperatures increased MDMA choice. Similar studies on these and other environmental variables will play a key role in future research to identify environmental mechanisms that may differentially affect the reinforcing strength of drugs and underlie vulnerability to or protection from drug addiction. 

#### 2.4.4. Effect of Subject-Related Biological Variables

 Intravenous drug choice procedures have been conducted in various species including, rats [62], squirrel monkeys [82], cynomolgus [92] and rhesus [29] macaques, and baboons [66]. However, there is substantial opportunity for more systematic research on the role of these and other subject-related variables, such as gender, genotype, or physiological state. To the best of our knowledge, there is only one example where a subject-related variable was manipulated in the context of drug choice. In this study, exogenous thyroid hormone was administered to induce a hyperthyroid state during MDMA versus food choice [94]. Although thyroid hormone treatment enhanced the thermogenic effects of MDMA, this treatment did not significantly alter MDMA choice. Moreover, drug choice studies can be expected to contribute important insights that might not be apparent from single-response or active/inactive-response procedures. Specifically, as has been emphasized repeatedly above, drug choice is strongly determined by factors that influence the reinforcing strength of alternative reinforcers. Consequently, it should be anticipated that some subject-related variables would have profound effects on drug choice by modulating the reinforcing strength of alternative reinforcers while producing little or no direct changes in the reinforcing strength of the drug. 

## 3. Implications and Future Directions

Research on the reinforcing effects of drugs has been slow to adopt concurrent schedules of reinforcement; however, this paper has argued that choice procedures can play a useful role in preclinical research on drug reinforcement and determinants of drugs use. There are still critical gaps in our knowledge, and there remains much intellectual space to be explored. One future direction might be the establishment of drug versus nondrug alternative choice procedures involving abused drugs other than the classical compounds cocaine and heroin. The degree to which drug choice can be maintained by other novel drug class such as benzodiazepines, cannabinoids, and nicotine remains to be elucidated. Another future direction might be to examine the impact of nondrug alternative reinforcers other than food. Food is an easy alternative reinforcer to control in preclinical laboratories, but there are certainly other nondrug reinforcers (e.g., access to receptive mate or social interactions) that are available as research tools that have yet to be fully explored. As one example of a choice procedure using a nondrug alternative reinforcer other than food, one intriguing study examined effects of putative anorectic drugs on choice between food and visual access to a room containing other monkeys [95]. This type of social reinforcer has yet to be manipulated in studies of drug choice. Finally, a third potential future direction is the integration of drug reinforcers into decision-making “choice” tasks commonly used to assess cognitive function. For example, impaired delay discounting is a cognitive trait commonly linked to drug abuse [96, 97], and delay discounting is often assessed preclinically in assays that compare choice between a delayed high-magnitude reinforcer and an immediate low-magnitude reinforcer [98]. Strikingly, preclinical research with this type of assay has relied exclusively on food as the consequent stimulus. The introduction of drugs as reinforcers into delay discounting and other cognitive tasks may provide new insights into the relationship between cognitive function and drug use. Overall, the body of literature cited in this paper supports the notion that choice procedures can facilitate data interpretation by providing a rate-independent measure of drug reinforcement, improve concordance between preclinical and clinical studies in translational research, and provide experimental access to critical independent variables that influence drug choice and drug addiction in natural environments.

## Figures and Tables

**Table 1:** Summary of published manuscripts reporting on IV drug self-administration under concurrent-choice schedules. Columns show the primary drug option(s), the alternative reinforcer(s) (sometimes also a drug), the species in which studies were conducted, the primary effect examined in the study, and the reference. Numbers in parentheses show drug unit doses in mg/kg/injection.

#	Drug (dose in mg/kg/inj)	Alternative reinforcer	Species	Main effect examined	Ref.
1	Cocaine (0.05–0.1)	Cocaine (0.013–0.8)	Rhesus	Effect of drug dose	[9]
2	Cocaine (0.05–0.1)	Cocaine (0.05–0.1)	Rhesus	Effect of schedule type	[30]
3	Cocaine (0.05 or 0.1)	Cocaine (0.013–0.8)	Rhesus	Effect of drug dose	[34]
4	Cocaine (0.025–0.2)	Cocaine (0.025–0.2)	Rhesus	Effects of dose and schedule manipulations	[33]
5	Cocaine (0.03–0.3)	Cocaine (0.03–0.3)	Rhesus	Effect of infusion delay	[32]
6	Cocaine (0.025–0.05)	Cocaine (0.025–0.05) Food pellet	Rhesus	Effect of reinforcement delay	[37]
7	Cocaine (0.05–0.2)	Cocaine (0.05–0.2)	Rhesus	Effect of reinforcement probability	[38]
8	Cocaine (0.025–0.1)	Cocaine (0.025–0.1)	Rhesus	Application of generalized matching law	[40]
9	Cocaine (0.025–0.1)	Cocaine (0.025–0.1)	Rhesus	Effect of schedule type	[39]
10	Cocaine (0.1–0.75)	Cocaine (0.1–0.75)	Rhesus	Effect of punishment (electric shock)	[71]
11	Cocaine (0.3–1)	Cocaine (0.3–1)	Rat	Effect of infusion rate	[35]
12	Nicotine (0.015)	Nicotine (0.015)	Rat	Effect of infusion rate	[36]
13	Cocaine (0.05) Alfentanil (0.001–0.004) Methohexital (0.25–0.5)	Cocaine (0.05) Alfentanil (0.001–0.004) Methohexital (0.25–0.5)	Rhesus	Application of generalized matching law	[41]
14	Cocaine (0.1–0.056) Remifentanil Methohexital (0.32)	Cocaine (0.1–0.056) Remifentanil (0.0001–0.00003) Methohexital (0.32)	Rhesus	Application of generalized matching law	[42]
15	Cocaine (0.05–1.5)	Cocaine (0.1–1.5) Methylphenidate (0.075–0.07) Diethylpropion (0.5–1)	Rhesus	Effect of various pharmacological and environmental manipulations	[31]
16	Cocaine (0.05–1.5)	Methylphenidate (0.075–0.7)	Rhesus	Drug versus drug preference	[10]
17	Cocaine (0.05–0.2)	d,l-Cathinone (0.05–0.2)	Rhesus	Drug versus drug preference	[47]
18	Cocaine (0–0.1)	Procaine (0.4–1.6)	Rhesus	Drug versus drug preference	[46]
19	Cocaine (0.01–0.03)	PTT (0.01–0.03)	Rhesus	Drug versus drug preference	[50]
20	Cocaine (0.01–0.03)	Remifentanil (0.0001–0.0003)	Rhesus	Behavioral economic analysis of choice	[51]
21	Cocaine (0.03)	Remifentanil (0.00003)	Rhesus	Effect of chronic morphine administration and withdrawal on drug choice	[78]
22	Cocaine (0.267 or 0.8)	Nicotine (8–75)	Rat	Drug versus drug preference	[49]
23	Cocaine (0.4)	Heroin (0.025)	Rat	Effect of home cage environment	[52]
24	Secobarbital (18–100 mg) Chlordiazepoxide (5–20 mg)	Saline Secobarbital	Rhesus	Effect of dependence and withdrawal	[29]
25	Cocaine (0.1)	Cocaine (0.01–0.56) Remifentanil (0.00003–0.003) Cocaine + Remifentanil	Rhesus	Effect of drug mixtures on drug choice	[69]
26	Cocaine (0.038–3)	Heroin (0.025–0.05) Cocaine + Heroin	Rat	Effect of drug mixtures on drug choice	[68]
27	Cocaine (0.8)	Cocaine (0.267–2.4) SKF82958 (0.003–0.03) (+)-PHNO (0.001–0.01) SKF82958 + (+)-PHNO	Rat	Effect of drug mixtures on drug choice	[48]
28	Cocaine (0.05–0.1)	Cocaine (0.05–0.1) + histamine (0.00037–0.0005)	Rhesus	Effect of punishment (IV histamine) delay on drug choice	[73]
29	Cocaine (0.3)	Food pellet	Rhesus	First study of cocaine versus food choice	[53]
30	Cocaine (0.03–1)	Food pellet	Rhesus	Effect of response requirement	[58]
31	Cocaine (0.03–1)	Food pellet	Rhesus	Effect of food availability conditions	[59]
32	Cocaine (0.05–0.4)	Food pellet	Rhesus	Behavioral economic analysis of choice	[99]
33	Cocaine (0.05–0.2)	Food pellet	Rhesus	Behavioral economic analysis of choice	[100]
34	Cocaine (0.025–0.05)	Food pellet	Rhesus	Application of generalized matching law	[101]
35	Cocaine (0.0032–0.32)	Food pellet	Rhesus	Effect of dose and cocaine pretreatment	[54]
36	Cocaine (0.33)	Food pellet or water	Rat	Effect of alternative reinforcer on drug choice	[63]
37	Cocaine (0.25)	Glucose/Saccharin solution	Rat	Effect of alternative reinforcer on drug choice	[62]
38	Cocaine (0.25–1.5)	Saccharin or Sucrose	Rat	Effect of sweet solutions on drug choice	[65]
39	Cocaine (0.1–0.3)	Food pellet	Rhesus	Effect of chronic lithium treatment	[86]
40	Cocaine (0.05–0.3)	Food pellet	Rhesus	Effect of chronic antipsychotic treatment	[85]
41	Cocaine (0.0032–0.1)	Food pellet	Rhesus	Effect of monoamine releasers with varying selectivity for dopamine versus serotonin	[17]
42	Cocaine (0.0032–0.1)	Food pellet	Rhesus	Effect of various pharmacological and environmental manipulations	[55]
43	Cocaine (0.0032–0.1)	Food pellet	Rhesus	Effect of chronic kappa opioid treatment	[84]
44	Cocaine (0.0032–0.1)	Food pellet	Rhesus	Effect of chronic methadone treatment	[18]
45	Cocaine (0.003–0.1)	Food pellet 10% Sweet Condensed milk	Rhesus Squirrel monkey	Effect of acute and chronic aripiprazole treatment	[93]
46	Cocaine (0.003–0.03)	Food pellet	Cynomolgus	Effect of 8-OH-DPAT treatment	[83]
47	Cocaine (0-1)	Ensure liquid food	Rat	Effect of acute and chronic aripiprazole treatment	[61]
	Cocaine (0-1)	Ensure liquid food	Rat	Effect of amphetamine treatment and environmental manipulations	[81]
48	Cocaine (0.25)	Saccharin	Rat	Effect of diazepam treatment	[82]
49	Cocaine (0.0032–0.1)	Food pellet	Rhesus	Effect of punishment (IV histamine)	[72]
50	Cocaine (0.0032–0.1)	Food pellet	Rhesus	Effect of exposure to and withdrawal from extended cocaine access	[80]
51	Cocaine (0.003–0.03)	Food pellet	Cynomolgus	Effect of social hierarchy	[91]
52	Cocaine (0.003–0.1)	Food pellet	Cynomolgus	Effect of social hierarchy and environmental stimuli	[56]
53	Cocaine (0.03–0.56) Procaine (1–10)	Food pellet	Rhesus	Effect of drug type and food reinforcer magnitude	[57]
54	Cocaine (0.003–0.3) Methylphenidate (0.003–0.1) Amphetamine (0.003–0.1) Atomoxetine (0.01–0.3) Desipramine (0.3–1)	Food pellet	Rhesus	Effects of drug type on drug versus food choice	[102]
55	Cocaine (0.0032–0.1) Heroin (0.0032–0.1) Cocaine + Heroin	Food pellet	Rhesus	Effects of drug mixtures on drug choice	[67]
56	Cocaine (0–0.1)	Food pellet	Rhesus	Reinstatement of cocaine choice by dopaminergic compounds	[103]
57	Heroin (0.1)	Food	Baboon	Effect of economic conditions	[60]
58	Heroin (0.055–0.83)	Food pellet ± Heroin (0.055–0.83)	Baboon	Various pharmacological and environmental manipulations	[66]
59	Heroin (0.32–0.96)	Food pellet	Baboon	Effect of methadone, naloxone treatment	[74]
60	Heroin (0.32 or 1)	Food pellet	Baboon	Effect of morphine, naloxone, secobarbital	[75]
61	Heroin (0.0032–0.1) Heroin + SNC80	Food pellet	Rhesus	Effect of drug mixtures on drug choice	[70]
62	Heroin (0.0032–0.1)	Food pellet	Rhesus	Effect of methadone, buprenorphine, naloxone treatment in nondependent and opioid-dependent monkeys	[76]
63	Heroin (0.0032–0.1)	Food pellet	Rhesus	Effect of morphine, amphetamine, clonidine, antalarmin, norbinaltorphimine treatment in opioid-dependent monkeys	[79]
64	MDMA (0.03–0.3)	Food pellet	Rhesus	Effect of ambient temperature	[92]
65	MDMA (0.03–0.3)	Food pellet	Rhesus	Effect of thyroid hormone levels	[94]
66	Methamphetamine (0.06)	Food pellet	Rat	Drug versus food preference	[64]

## References

[1] Ator NA, Griffiths RR (2003). Principles of drug abuse liability assessment in laboratory animals. *Drug and Alcohol Dependence*.

[2] Carter LP, Griffiths RR (2009). Principles of laboratory assessment of drug abuse liability and implications for clinical development.. *Drug and Alcohol Dependence*.

[3] Skinner BF (1938). *The Behavior of Organisms*.

[4] Ferster CB, Skinner BF (1957). *Schedules of Reinforcement*.

[5] Weeks JR (1962). Experimental morphine addiction: method for automatic intravenous injections in unrestrained rats. *Science*.

[6] Weeks JR, Collins RJ (1964). Factors affecting voluntary morphine intake in self-maintained addicted rats. *Psychopharmacologia*.

[7] Young A, Herling S, Goldberg S, Stolerman I (1986). Drugs as reinforcers: studies in laboratory animals. *Behavioral Analysis of Drug Dependence*.

[8] Katz J, Leibman J, Cooper S (1989). Drugs as reinforcers: pharmacological and behavioral factorsin. *The Neuropharmacological Basis of Reward*.

[9] Iglauer C, Woods JH (1974). Concurrent performances: reinforcement by different doses of intravenous cocaine in rhesus monkeys. *Journal of the Experimental Analysis of Behavior*.

[10] Johanson CE, Schuster CR (1975). A choice procedure for drug reinforcers: cocaine and methylphenidate in the rhesus monkey. *Journal of Pharmacology and Experimental Therapeutics*.

[11] Mello NK, Negus SS (1996). Preclinical evaluation of pharmacotherapies for treatment of cocaine and opioid abuse using drug self-administration procedures. *Neuropsychopharmacology*.

[12] Zernig G, Wakonigg G, Madlung E (2004). Do vertical shifts in dose-response rate-relationships in operant conditioning procedures indicate ‘sensitization’ to ‘drug wanting’?. *Psychopharmacology*.

[13] Meisch R, Lemaire G, Van Haaren F (1993). Drug self-administration. *Methods In Behavioral Pharmacology*.

[14] Deneau G, Yanagita T, Seevers MH (1969). Self-administration of psychoactive substances by the monkey-a measure of psychological dependence. *Psychopharmacologia*.

[15] Kelleher RT, Goldberg SR (1975). General introduction: control of drug taking behavior by schedules of reinforcement. *Pharmacological Reviews*.

[16] Pickens R, Thompson T (1968). Cocaine-reinforced behavior in rats: effects of reinforcement magnitude and fixed-ratio size.. *Journal of Pharmacology and Experimental Therapeutics*.

[17] Banks ML, Blough BE, Negus SS (2011). Effects of monoamine releasers with varying selectivity for releasing dopamine/norepinephrine versus serotonin on choice between cocaine and food in rhesus monkeys. *Behavioural Pharmacology*.

[18] Negus SS, Mello NK (2004). Effects of chronic methadone treatment on cocaine- and food-maintained responding under second-order, progressive-ratio and concurrent-choice schedules in rhesus monkeys. *Drug and Alcohol Dependence*.

[19] Negus SS, Banks ML, Glennon RA, Young R (2011). Making the right choice: lessons from drug discrimination for research on drug reinforcement and drug self-administration. *Drug DiscrimInation: Applications To MedicInal Chemistry and Drug Studies*.

[20] Spragg SDS (1940). Morphine addiction in chimpanzees. *Comparative Psychology Monographs*.

[21] Nichols JR, Davis WM (1959). Drug addiction. II. Variation of addiction. *Journal of the American Pharmacists Association*.

[22] Heyman GM (1993). Ethanol regulated preference in rats. *Psychopharmacology*.

[23] Comer SD, Ashworth JB, Foltin RW, Johanson CE, Zacny JP, Walsh SL (2008). The role of human drug self-administration procedures in the development of medications. *Drug and Alcohol Dependence*.

[24] Haney M, Spealman R (2008). Controversies in translational research: drug self-administration. *Psychopharmacology*.

[25] Heyman GH (2009). *Addiction: A Disorder of Choice*.

[26] Hernstein R, Prelec D, Loewenstein G, Elster J (1992). A theory of addiction. *Choice Over Time*.

[27] American Psychiatric Association (2000). *Diagnostic and Statistical Manual of Mental Disorders*.

[28] http://www.dsm5.org/proposedrevision/Pages/SubstanceUseandAddictiveDisorders.aspx.

[29] Findley JD, Robinson WW, Peregrino L (1972). Addiction to secobarbital and chlordiazepoxide in the rhesus monkey by means of a self-infusion preference procedure. *Psychopharmacologia*.

[30] Iglauer C, Llewellyn ME, Woods JH (1975). Concurrent schedules of cocaine injection in rhesus monkeys: dose variations under independent and non independent variable interval procedures. *Pharmacological Reviews*.

[31] Johanson CE (1975). Pharmacological and environmental variables affecting drug preference in rhesus monkeys. *Pharmacological Reviews*.

[32] Anderson KG, Woolverton WL (2003). Effects of dose and infusion delay on cocaine self-administration choice in rhesus monkeys. *Psychopharmacology*.

[33] Anderson KG, Woolverton WL (2004). Dose and schedule determinants of cocaine choice under concurrent variable-interval schedules in rhesus monkeys. *Psychopharmacology*.

[34] Llewellyn ME, Iglauer C, Woods JH (1976). Relative reinforcer magnitude under a nonindependent concurrent schedule of cocaine reinforcement in rhesus monkeys. *Journal of the Experimental Analysis of Behavior*.

[35] Schindler CW, Panlilio LV, Thorndike EB (2009). Effect of rate of delivery of intravenous cocaine on self-administration in rats. *Pharmacology Biochemistry and Behavior*.

[36] Sorge RE, Clarke PBS (2009). Rats self-administer intravenous nicotine delivered in a novel smoking-relevant procedure: effects of dopamine antagonists. *Journal of Pharmacology and Experimental Therapeutics*.

[37] Woolverton WL, Anderson KG (2006). Effects of delay to reinforcement on the choice between cocaine and food in rhesus monkeys. *Psychopharmacology*.

[38] Woolverton WL, Rowlett JK (1998). Choice maintained by cocaine or food in monkeys: effects of varying probability of reinforcement. *Psychopharmacology*.

[39] Woolverton WL (1996). Intravenous self-administration of cocaine under concurrent VI schedules of reinforcement. *Psychopharmacology*.

[40] Woolverton WL, Alling K (1999). Choice under concurrent VI schedules: comparison of behavior maintained by cocaine or food. *Psychopharmacology*.

[41] Anderson KG, Woolverton WL (2000). Concurrent variable-interval drug self-administration and the generalized matching law: a drug-class comparison. *Behavioural Pharmacology*.

[42] Koffarnus MN, Woods JH (2008). Quantification of drug choice with the generalized matching law in rhesus monkeys. *Journal of the Experimental Analysis of Behavior*.

[43] Hursh SR (1980). Economic concepts for the analysis of behavior. *Journal of the Experimental Analysis of Behavior*.

[44] Bickel WK, DeGrandpre RJ, Higgins ST (1995). The behavioral economics of concurrent drug reinforcers: a review and reanalysis of drug self administration research. *Psychopharmacology*.

[45] Hursh SR, Bauman R, Green L, Kagel J (1987). The behavioral analysis of demand. *Advances In Behavioral Economics*.

[46] Johanson CE, Aigner T (1981). Comparison of the reinforcing properties of cocaine and procaine in rhesus monkeys. *Pharmacology Biochemistry and Behavior*.

[47] Woolverton WL, Johanson CE (1984). Preference in rhesus monkeys given a choice between cocaine and d,l-cathinone.. *Journal of the Experimental Analysis of Behavior*.

[48] Manzardo AM, Del Rio JA, Stein L, Belluzzi JD (2001). Rats choose cocaine over dopamine agonists in a two-lever self-administration preference test. *Pharmacology Biochemistry and Behavior*.

[49] Manzardo AM, Stein L, Belluzzi JD (2002). Rats prefer cocaine over nicotine in a two-lever self-administration choice test. *Brain Research*.

[50] Lile JA, Morgan D, Birmingham AM (2002). The reinforcing efficacy of the dopamine reuptake inhibitor 2*β*-propanoyl-3*β*-(4-tolyl)-tropane (PTT) as measured by a progressive-ratio schedule and a choice procedure in rhesus monkeys. *Journal of Pharmacology and Experimental Therapeutics*.

[51] Wade-Galuska T, Winger G, Woods JH (2007). A behavioral economic analysis of cocaine and remifentanil self-administration in rhesus monkeys. *Psychopharmacology*.

[52] Caprioli D, Celentano M, Dubla A, Lucantonio F, Nencini P, Badiani A (2009). Ambience and drug choice: cocaine- and heroin-taking as a function of environmental context in humans and rats. *Biological Psychiatry*.

[53] Aigner TG, Balster RL (1978). Choice behavior in rhesus monkeys: cocaine versus food. *Science*.

[54] Paronis CA, Gasior M, Bergman J (2002). Effects of cocaine under concurrent fixed ratio schedules of food and IV drug availability: a novel choice procedure in monkeys. *Psychopharmacology*.

[55] Negus SS (2003). Rapid assessment of choice between cocaine and food in rhesus monkeys: effects of environmental manipulations and treatment with d-amphetamine and flupenthixol. *Neuropsychopharmacology*.

[56] Czoty PW, Nader MA (2012). Individual differences in the effects of environmental stimuli on cocaine choice in socially housed male cynomolgus monkeys. *Psychopharmacology*.

[57] Nader MA, Woolverton WL (1991). Effects of increasing the magnitude of an alternative reinforcer on drug choice in a discrete-trials choice procedure. *Psychopharmacology*.

[58] Nader MA, Woolverton WL (1992). Effects of increasing response requirement on choice between cocaine and food in rhesus monkeys. *Psychopharmacology*.

[59] Nader MA, Woolverton WL (1992). Choice between cocaine and food by rhesus monkeys: effects of conditions of food availability. *Behavioural Pharmacology*.

[60] Elsmore TF, Fletcher GV, Conrad DG, Sodetz FJ (1980). Reduction of heroin intake in baboons by an economic constraint. *Pharmacology Biochemistry and Behavior*.

[61] Thomsen M, Fink-Jensen A, Woldbye DPD (2008). Effects of acute and chronic aripiprazole treatment on choice between cocaine self-administration and food under a concurrent schedule of reinforcement in rats. *Psychopharmacology*.

[62] Carroll ME, Lac ST, Nygaard SL (1989). A concurrently available nondrug reinforcer prevents the acquisition or decreases the maintenance of cocaine-reinforced behavior. *Psychopharmacology*.

[63] Dworkin SI, Mirkis S, Smith JE (1990). Reinforcer interactions under concurrent schedules of food, water, and intravenous cocaine. *Behavioural Pharmacology*.

[64] Ping A, Kruzich PJ (2008). Concurrent access to sucrose pellets decreases methamphetamine-seeking behavior in Lewis rats. *Pharmacology Biochemistry and Behavior*.

[65] Lenoir M, Serre F, Cantin L, Ahmed SH (2007). Intense sweetness surpasses cocaine reward. *PloS one*.

[66] Wurster RM, Griffiths RR, Findley JD, Brady JV (1977). Reduction of heroin self-administration in baboons by manipulation of behavioral and pharmacological conditions. *Pharmacology Biochemistry and Behavior*.

[67] Negus SS (2005). Interactions between the reinforcing effects of cocaine and heroin in a drug-vs-food choice procedure in rhesus monkeys: a dose-addition analysis. *Psychopharmacology*.

[68] Ward SJ, Morgan D, Roberts DCS (2005). Comparison of the reinforcing effects of cocaine and cocaine/heroin combinations under progressive ratio and choice schedules in rats. *Neuropsychopharmacology*.

[69] Freeman KB, Woolverton WL (2011). Self-administration of cocaine and remifentanil by monkeys: choice between single drugs and mixtures. *Psychopharmacology*.

[70] Stevenson GW, Folk JE, Rice KC, Negus SS (2005). Interactions between *δ* and *μ* opioid agonists in assays of schedule-controlled responding, thermal nociception, drug self-administration, and drug versus food choice in rhesus monkeys: studies with SNC80 [(+)-4-[(*α*R)-*α*-((2S,5R)-4-allyl-2,5-dimethyl-1-piperazinyl) -3-methoxybenzyl]-N,N-diethylbenzamide] and heroin. *Journal of Pharmacology and Experimental Therapeutics*.

[71] Johanson CE (1977). The effects of electric shock on responding maintained by cocaine injections in a choice procedure in the rhesus monkey. *Psychopharmacology*.

[72] Negus SS (2005). Effects of punishment on choice between cocaine and food in rhesus monkeys. *Psychopharmacology*.

[73] Woolverton WL, Freeman KB, Myerson J, Green L (2012). Suppression of cocaine self-administration in monkeys: effects of delayed punishment. *Psychopharmacology*.

[74] Griffiths RR, Wurster RM, Brady JV (1975). Discrete trial choice procedure: effects of naloxone and methadone on choice between food and heroin. *Pharmacological Reviews*.

[75] Griffiths RR, Wurster RM, Brady JV (1981). Choice between food and heroin: effects of morphine, naloxone, and secobarbital.. *Journal of the Experimental Analysis of Behavior*.

[76] Negus SS (2006). Choice between heroin and food in nondependent and heroin-dependent rhesus monkeys: effects of naloxone, buprenorphine, and methadone. *Journal of Pharmacology and Experimental Therapeutics*.

[77] Negus SS, Dean R, Bilsky EJ, Negus SS (2009). Opioid antagonist effects in animal models related to opioid abuse: drug discrimination and drug self-administration. *Opiate RecepTors and Antagonists: From Bench To Clinic*.

[78] Wade-Galuska T, Galuska CM, Winger G (2011). Effects of daily morphine administration and deprivation on choice and demand for remifentanil and cocaine in rhesus monkeys. *Journal of the Experimental Analysis of Behavior*.

[79] Negus SS, Rice KC (2009). Mechanisms of withdrawal-associated increases in heroin self-administration: pharmacologic modulation of heroin vs food choice in heroin-dependent rhesus monkeys. *Neuropsychopharmacology*.

[80] Banks ML, Negus SS (2010). Effects of extended cocaine access and cocaine withdrawal on choice between cocaine and food in rhesus monkeys. *Neuropsychopharmacology*.

[81] Thomsen M, Barrett A, Negus SS, Caine SB Cocaine-food choice rats: environmental manipulations and effects of amphetamine.

[82] Augier E, Vouillac C, Ahmed SH (2012). Diazepam promotes choice of abstinence in cocaine self-administering rats. *Addiction Biology*.

[83] Czoty PW, McCabe C, Nader MA (2005). Effects of the 5-HT1A agonist (±)-8-hydroxy-2-(di-n- propylamino)tetralin (8-OH-DPAT) on cocaine choice in cynomolgus monkeys. *Behavioural Pharmacology*.

[84] Negus SS (2004). Effects of the kappa opioid agonist U50,488 and the kappa opioid antagonist nor-binaltorphimine on choice between cocaine and food in rhesus monkeys. *Psychopharmacology*.

[85] Woolverton WL, Balster RL (1981). Effects of antipsychotic compounds in rhesus monkeys given a choice between cocaine and food. *Drug and Alcohol Dependence*.

[86] Woolverton WL, Balster RL (1979). The effects of lithium on choice between cocaine and food in the rhesus monkey. *Communications in Psychopharmacology*.

[87] Vocci FJ (2007). Commentary: can replacement therapy work in the treatment of cocaine dependence? and what are we replacing anyway?. *Addiction*.

[88] Greenwald MK, Lundahl LH, Steinmiller CL (2010). Sustained release d-amphetamine reduces cocaine but not speedball-seeking in buprenorphine-maintained volunteers: a test of dual-agonist pharmacotherapy for cocaine/heroin polydrug abusers. *Neuropsychopharmacology*.

[89] Rush CR, Stoops WW, Sevak RJ, Hays LR (2010). Cocaine choice in humans during D-amphetamine maintenance. *Journal of Clinical Psychopharmacology*.

[90] Walsh SL, Geter-Douglas B, Strain EC, Bigelow GE (2001). Enadoline and butorphanol: evaluation of *κ*-agonists on cocaine pharmacodynamics and cocaine self-administration in humans. *Journal of Pharmacology and Experimental Therapeutics*.

[91] Czoty PW, McCabe C, Nader MA (2005). Assessment of the relative reinforcing strength of cocaine in socially housed monkeys using a choice procedure. *Journal of Pharmacology and Experimental Therapeutics*.

[92] Banks ML, Sprague JE, Czoty PW, Nader MA (2008). Effects of ambient temperature on the relative reinforcing strength of MDMA using a choice procedure in monkeys. *Psychopharmacology*.

[93] Bergman J (2008). Medications for stimulant abuse: agonist-based strategies and preclinical evaluation of the mixed-action D2 partial agonist aripiprazole (Abilify®). *Experimental and Clinical Psychopharmacology*.

[94] Banks ML, Czoty PW, Sprague JE, Nader MA (2008). Influence of thyroid hormones on 3,4-methylenedioxymethamphetamine-induced thermogenesis and reinforcing strength in monkeys. *Behavioural Pharmacology*.

[95] Corwin RL, Schuster CR (1993). Anorectic specificity as measured in a choice paradigm in rhesus monkeys. *Pharmacology Biochemistry and Behavior*.

[96] Marsch LA, Bickel WK (2001). Toward a behavioral economic understanding of drug dependence: delay discounting processes. *Addiction*.

[97] Monterosso J, Piray P, Luo S (2012). Neuroeconomics and the study of addiction. *Biological Psychiatry*.

[98] Winstanley CA (2011). The utility of rat models of impulsivity in developing pharmacotherapies for impulse control disorders. *British Journal of Pharmacology*.

[99] Woolverton WL, English JA, Weed MR (1997). Choice between cocaine and food in a discrete-trials procedure in monkeys: a unit price analysis. *Psychopharmacology*.

[100] Woolverton WL, English JA (1997). Further analysis of choice between cocaine and food using the unit price model of behavioral economics. *Drug and Alcohol Dependence*.

[101] Anderson KG, Velkey AJ, Woolverton WL (2002). The generalized matching law as a predictor of choice between cocaine and food in rhesus monkeys. *Psychopharmacology*.

[102] Gasior M, Bergman J, Kallman MJ, Paronis CA (2005). Evaluation of the reinforcing effects of monoamine reuptake inhibitors under a concurrent schedule of food and i.v. drug delivery in rhesus monkeys. *Neuropsychopharmacology*.

[103] Gasior M, Paronis CA, Bergman J (2004). Modification by dopaminergic drugs of choice behavior under concurrent schedules of intravenous saline and food delivery in monkeys. *Journal of Pharmacology and Experimental Therapeutics*.

